# Mr. John William Adrian

**Published:** 1910-08-13

**Authors:** 


### Obituary.

Mr. John William Adrian died recently at his residence, Drogheda. He took the M.R.C.S. Eng. in 1858, and the L.K.Q.C.P. Ireland, in 1860, and was Poor Law medical officer for fifty years—ten years as dispensary medical officer of Stamullen district, in the Drogheda Union, and forty years as medical officer of the Drogheda Workhouse Hospital and Fever Hospital. He was very popular in Drogheda and the surrounding district, where he enjoyed a large practice. "Dr. Johnnie," as he was popularly known, remarks the *Journal* of the Irish Medical Association, came of a family of medical men. His father, the late Dr. William Edward Adrian, of Oldtown, County Dublin, was for many years medical officer of the Balrothery Workhouse Hospital. His brother, Dr. Edward Adrian, of Balbriggan, was a highly esteemed and successful practitioner until his death, a few years ago, while comparatively a young man. His grandfather, Surgeon William Adrian, of 40 Dawson Street, Dublin, was a well-known and eminent medical man in the stormy days of 1798, when he attended Lord Edward Fitzgerald and dressed his wounds, in a house in Thomas Street, Dublin. A keen sportsman in his younger days, Dr. John W. Adrian was a conspicuous figure at the meets of the Meath and Louth Hounds. He was for many years a member, and member of Council of the Irish Medical Association, and showed his practical interest in the working of the Association by his regular attendance at the meetings.

